# Wylie (W. Gill) on Dysmenorrhea and Chronic Endometritis

**Published:** 1894-06

**Authors:** 


WYLIE (W. GILL) ON DYSMENORRHEA AND CHRONIC ENDOMETRITIS.—Undue importance has been attached to retroversion, and other displacements as the direct cause of symptoms. Displacements simply complicate disease, and may give rise to pain, which, however, is due less to the displacement than to other conditions.

When the broad ligaments are thickened, and the tubes and ovaries diseased, pain will be experienced, as the subject assumes the upright position, even when there is no displacement from the downward pressure exerted upon the fixed uterus and diseased ovaries. When the uterus is healthy, displacement causes but little trouble.

If retroversion exist, next determine whether the case be complicated by disease of the endometrium, or by salpingitis.

If disease of the tubes and ovaries be present, the only procedure is their removal. We cannot treat disease of the Fallopian tubes by curetting and draining. We cannot reach the seat of disease except by laparotomy.

If no such complication exist, boro-glyceride tampons should be placed twice a week between the menstrual periods—but pessaries have no place in treatment.

If such treatment fail to effect involution and improved circulation, and the introduction of the sound cause bleeding, or pain, then douche, curette, and drain, making a simple application—never one that will destroy the mucous lining with its deep seated glands and follicles, leaving a scar that will do more harm than good in after life.

As to the curetting, the sharp instrument only is worthy of use. The blunt copper wire curette may turn up the granulations, but will not remove them, leaving behind a better nidus for sepsis than before the dilatation. When there are large pieces of tissue to be removed, blunt and rounded forceps are to be used.—*Internat. Jour. of Surg.*

